# Comparison of lipophilic and size-exclusion membranes: the effect of stirring and cyclodextrin in the donor compartment

**DOI:** 10.5599/admet.2753

**Published:** 2025-07-05

**Authors:** Petra Tőzsér, Szabina Kádár, Edina Szabó, Máté Dobó, Gergő Tóth, György T. Balogh, Péter Sóti, Bálint Sinkó, Enikő Borbás

**Affiliations:** 1 Department of Organic Chemistry and Technology, Faculty of Chemical Technology and Biotechnology, Budapest University of Technology and Economics, 3 Műegyetem Quay, H-1111, Budapest, Hungary; 2 Department of Pharmaceutical Chemistry, Faculty of Pharmaceutical Sciences, Semmelweis University, 9 Hőgyes Endre Street., H-1092, Budapest, Hungary; 3 Center for Pharmacology and Drug Research & Development, Semmelweis University, 26 Üllői Street., H-1085, Budapest, Hungary; 4 Department of Chemical and Environmental Process Engineering, Faculty of Chemical Technology and Biotechnology, Budapest University of Technology and Economics, 3 Műegyetem Quay., H-1111, Budapest, Hungary; 5 Lavet Pharmaceutical Ltd., 6 Batthyány Street., H-2143, Kistarcsa, Hungary; 6 Pion Inc., Billerica, 10 Cook Street, Massachusetts 01821, USA

**Keywords:** Unstirred water layer, membrane transport, solubility, supersaturation ratio, carvedilol

## Abstract

**Background and purpose:**

The effective transport of an active pharmaceutical ingredient across various membrane systems is critical for enhancing its bioavailability, especially in formulations involving solubilizing agents. This study aims to investigate the permeability differences of carvedilol (CAR) between lipophilic and size-exclusion membranes in the presence of hydroxypropyl-beta-cyclodextrin (HP-β-CD) using *in vitro* side-by-side diffusion cell assays.

**Experimental approach:**

Solubility and permeability assays confirmed that HP-β-CD significantly enhanced the solubility of CAR, while simultaneously decreasing its permeability, indicating an interplay between the two parameters.

**Key results:**

A mathematical model based on Fick’s first law of diffusion was developed to describe drug transport across the UWL, and generally through the UWL-membrane system, with a particular focus on the role of solubilizing agents.

**Conclusion:**

Results from both the UWL and membrane limited transport conditions demonstrated that the supersaturation ratio (SSR, defined as the ratio of the drug concentration present in solution to its thermodynamic solubility measured in exactly the same media) between donor and acceptor compartments is the real driving force of the transport, when the complexing agent and the drug- HP-β-CD complex does not penetrate the membrane or the permeation of the solubilizing additive through the membrane is relatively slow, so it does not affect the transport of the API substantially.

## Introduction

Over the last decade, the evaluation of intestinal drug permeation using *in vitro* systems has gained focus, leading to the development of numerous new cell-free permeation tools. Despite the great interest in intestinal tissue or cell layers to investigate the intestinal permeation potential of drug compounds, their application in industrial laboratories is strongly discouraged and complicated due to lengthy preparation steps. While cell-based systems are still preferred for assessing the apparent drug permeability coefficient (*P*_app_), there is a paradigm shift from the traditional cell- and tissue-based permeation barriers towards cell-free permeation barriers [1-8].

Transport through the gut wall *in vivo* and through the lipophilic membrane *in vitro* can be rate-limited by the transport through the membrane (epithelial cell membrane or lipid layer, respectively) or by diffusion through the so-called unstirred water layer (UWL). In lipophilic active pharmaceutical ingredients (APIs), cellular membrane permeation is very fast and permeation through the ca. 300 μm thick UWL on the apical side of the membrane becomes the rate-limiting step of the transport *in vivo* [9,10]. However, in an *in vitro* permeability assay with a planar membrane, the UWL can be up to 1500 to 4000 μm thick in non-stirred conditions. Without vigorous stirring, the UWL usually dominates the apparent permeability if its value exceeds 3 10^-5^ cm s^-1^. In case of cell- or tissue-based permeation barriers, the use of stirring negatively affects the viability of the membrane, therefore, vigorous stirring cannot be used, leading to UWL limited transport conditions even if it is not the case *in vivo*. For cell-free setups with efficient stirring, much thinner UWL can be created than the approximated thickness of the *in vivo* UWL [10]. Therefore, stirring such *in vitro* apparatuses is an important factor and must be investigated.

Artificial membranes based on filters coated with a phospholipid layer [11,12] are widely used in pharmaceutical development to mimic the *in vivo* composition of the epithelium layer in the gastrointestinal tract (GIT) due to their reasonably accurate predictive capabilities in case of the permeability of pure APIs and also in case of the permeability assessment of drug formulations [2,7,8,13]. Like all models, these also have some disadvantages, such as the lipids being stored frozen and potentially being chemically incompatible with biological gastrointestinal fluid [14] or with lipid formulations where the drug can be dissolved in a medium containing enzymes [15], which could affect the integrity of the lipophilic membrane.

Size-exclusion membranes are often referred to as dialysis membranes, where the pore sizes of the membrane are below 2 to 5 nm (or the molecular weight cut-off (MWCO) <14 kDa). Cellulose-acetate-based size-exclusion membranes are used extensively in pharmaceutical research to characterize drug-release kinetics from micro-disperse systems [16] and measure the particle size of aggregates, micelles, or complexes [17]. Furthermore, these membranes model the absorption of digested food in the intestines, excluding macromolecules and allowing only small digestive products to permeate [18]. Recent applications include the characterization of highly supersaturated solutions containing lipophilic drugs, demonstrating the reservoir effect of the drug-rich nanodroplets formed following liquid-liquid phase separation (LLPS) [19,20]. There is a growing interest in evaluating the applicability of size-exclusion membranes for assessing the intestinal permeation potential of different formulations of the same API; therefore, these membranes are also integrated into various side-by-side diffusion cell setups [2,3,21-24].

Size-exclusion membranes are pre-soaked in aqueous media to fill the pores. The transport of neutral and ionized molecules occurs through diffusion in the water-filled pores unless their hydrodynamic size limits their transport. Consequently, other structures present in the dissolution media, i.e., surfactants and cyclodextrins (CDs), may also reach the acceptor side [24]. While in the case of transport through a lipophilic membrane, there are two aqueous phases separated by an apolar organic phase, and only the neutral drug molecules partition from the donor aqueous phase into the organic phase, then partition again into the acceptor phase after diffusing through the lipophilic phase. If the transport through the aqueous boundary layer adjacent to the membrane towards the membrane surface is the rate-limiting step of the transport, then the term UWL-limited transport is used. In UWL-limited transport, the effective concentration gradient across the membrane is diminished because of slow diffusion in the unstirred water layer, particularly in the case of highly lipophilic drugs. In the case of size-exclusion membranes, a different permeation process occurs, as the membrane does not contain lipids. The donor and acceptor sides are filled with the same buffer solution, measuring transport speed through an aqueous medium. Consequently, for size-exclusion membranes, both the UWL and the membrane permeation involve aqueous diffusion, with no diffusion through a lipophilic phase. However, these two aqueous environments may differ significantly due to the drug's molecular hydration and potential interaction with the filter material. In the case of the size-exclusion membrane, in addition to the presence of UWL [25], it is also necessary to distinguish between the two cases when the excipient passes through the membrane and when it does not.

Dissolution-permeation setups may contain many different artificial membranes. In general, they demonstrate better discrimination of formulations compared to simple dissolution setups, enabling the study of the effects of formulation additives on dissolution and membrane transport. Solubilizing additives, such as surfactants and complexing agents (CDs), generally enhance the solubility of the APIs while reducing free drug concentration, decreasing the flux across the membrane [26]. Moreover, the decrease in the apparent permeability was found to be proportional to the solubility enhancement achieved by the complexing agent [27,28]. This interplay between solubility and permeability was first demonstrated in the rat perfusion model and Parallel Artificial Membrane Permeability Assay [11] (PAMPA) membranes [29]. Later, it was shown that a similar phenomenon can be captured using cellulose membranes [19,22,23,30,31] when only the free drug can cross the size-exclusion membrane; however, most of these membranes cannot distinguish the ionized and the neutral form of the drug. Studies were also conducted to evaluate how the composition and type of polymer can affect the dissolution and permeation of amorphous solid dispersions implementing size-exclusion membranes [19,22,32].

In conclusion, the use of cellulose-based membranes as a simple surrogate for Caco-2 or PAMPA membranes in dissolution-permeation assays of drug formulations presenting solubility-permeability interplay has already been published several times in the literature; however, a thorough analysis is needed to understand the underlying mechanism and uncover the limitations of such application. For that reason, this study aims to investigate whether size-exclusion membranes can be an alternative for lipophilic membranes in the case of studying drug formulations of the same API and what the advantages and limitations are. Therefore, this paper aims to compare the simplest lipophilic (PVDF filter impregnated with *n*-dodecane) and size-exclusion membranes (molecular weight cut-offs of 1 kDa and 6 to 8 kDa) on theoretical grounds, studying the driving force of membrane transport. Practical aspects are also considered to see what assay conditions might be used for the membranes to see a similar effect when adding a solubilizing additive. Carvedilol, a non-selective β-receptor blocker, was chosen as the model API, while the model solubilizing additive with known size and attributes, hydroxypropyl-β-cyclodextrin (HP-β-CD), was chosen, which shows the highest complexation efficiency (CE) with CAR among the cyclodextrins [33-35].

## Experimental

### Materials

**Figure 1.**
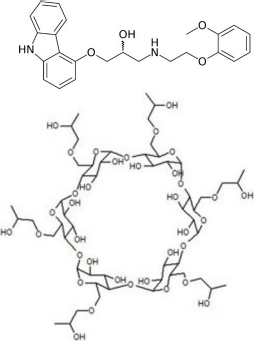
The structural formula of CAR [40] and hydroxypropyl-beta-cyclodextrin [41]

Carvedilol Form II (CAR, a BCS Class II, poorly water-soluble but well permeable, monovalent weak base with a p*K*_a_ of 7.78 ± 0.04 [36,37] (at 37 °C, *I* = 0.15), 406.482 g mol^-1^, structure shown in Figure 1) was purchased from Sigma-Aldrich Co. LLC. (St. Louis, MO, USA). From the commercially available Form II, the thermodynamically stable Form I was prepared in-house based on patents and literature data [38,39] (main parameters: Form II suspended in ethyl acetate at 50°C, crystal seeding and stirring for 3 h, cooling for room temperature) and was verified by X-Ray powder diffraction (XRPD) and Raman-spectroscopy. HP-β-CD, a doughnut-shaped molecule consisting of seven alpha-D-glucopyranose units with an internal cavity known as a solubilizing agent (DS: 4.34, average molecular weight 1391 g mol^-1^, structure shown in Figure 1), was obtained from Janssen (Beerse, Belgium).

Methanol was purchased from Thomasker (Budapest, Hungary), Prisma^TM^ HT universal buffer concentrate (suggested working pH range between 3 and 10), which is a commercially available ready-to-use buffer solution was obtained from Pion Inc. (Billerica, MA, USA) and *n*-dodecane from Sigma-Aldrich (St. Louis, Missouri, USA). 1 and 6 to 8 kDa MWCO (the MWCO of the membrane is determined as the molecular weight of the smallest solute which is at least 90 % retained during the test (the smallest solute for which the permeation is 10 % or less) regenerated cellulose size-exclusion membrane were purchased from Repligen (Boston, MA, USA). Polyvinylidene fluoride (PVDF) sheet with 0.45 μm pore size was obtained from Pion Inc. (Billerica, MA, USA).

### Determination of the limiting step of membrane transport

The *in vitro* side-by-side diffusion cell assays were performed using μFLUX™ apparatus (Pion Inc., Billerica MA, USA) to determine UWL thickness. It consists of a donor and an acceptor chamber (20 mL volumes of plain buffer or buffer with HP-β-CD on the donor side and plain buffer on the acceptor side) separated by an artificial membrane selected to be a lipophilic membrane (0.45 μm pore size PVDF sheet impregnated with 25 μL *n*-dodecane), 1 or 6 kDa size-exclusion membrane. The transport of CAR across the different membranes was investigated at different pH values and stirring speeds (Table 1).

**Table 1.** Parameters of the UWL thickness measurement in the case of lipophilic membrane with different pH values and stirring rates

Membrane	pH	Stirring rates, rpm	Content of neutral form of Carvedilol, %
Lipophilic	10.0	0; 25; 50; 100; 250; 400; 600	99.4
	9.5	25; 50; 100; 250	98.0
	7.0	25; 50; 100; 250	13.7
	6.0	25; 50; 100; 250	1.6
	5.0	25; 50; 100; 250	0.2
Size-exclusion	10.0	0; 100; 250; 400	99.4
	6.0	250	1.6

A lipophilic membrane (PVDF sheet, 0.45 μm pore size) impregnated with 25 μL *n*-dodecane was placed in the membrane holder connecting the donor and acceptor cells. 18 mL of Prisma buffer, adjusted to the appropriate pH, was pipetted into the donor and acceptor sides. The appropriate amount of the 3 mg mL^-1^ methanol stock solution of CAR was added to the donor side. The target concentrations were 20, 10, 50 and 50 mg mL^-1^ for pH 10, pH 9.5, pH 7, and pH 6, respectively. Methanol was pipetted to the acceptor side in the same amount as that added to the donor side. To ensure that the starting solution was homogeneous in all cases, both the donor and acceptor sides were stirred at 250 rpm for 1 min, and then the specified stirring speed (Table 1) was set. The concentration of CAR on both the donor and acceptor sides was measured in real-time using UV probes. The measurements were performed at 37 °C. The spectra obtained were evaluated by the second derivative method between 292 and 300 nm, and the apparent permeability (*P*_app_) was calculated [9] according to Equation (1) as follows:



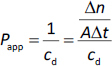

(1)


where flux (*J*) of a drug through the membrane is defined as the amount (*n*) of the drug crossing a unit area (*A*) perpendicular to its flow per unit time (*t*). *c*_d_ is the concentration of the drug at the donor side. The membrane permeability of the unbound (u) and unionized drug (*P*_membrane, lipophilic,u,0_) was determined using the Gutknecht method [42-45] based on the measured apparent permeability.

Quasi-Equilibrium model used to determine the UWL permeability of the drug in the presence and absence of cyclodextrin [46]:

The assumptions of the analysis: a) quasi-equilibrium conditions, b) 1:1 stoichiometry between CAR/ HP-β-CD complex, c) only the free drug partitions and diffuses into the membrane, but not the CAR/ HP-β-CD complex, d) the presence of HP-β-CD does not affect the membrane thickness, e) only the free drug encounters a significant unstirred water layer thickness, but the unstirred water layer thickness is negligible for the CAR/ HP-β-CD complex.

*P*_UWL_ and *P*_membrane_ were calculated using Equations (2) and (3) as follows:



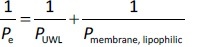

(2)




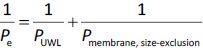

(3)


where *P*_membrfane, lipophilic_ and *P*_membrane, size-exclusion_ are the membrane permeability of the drug molecules for lipophilic and size-exclusion membranes, and *P*_UWL_ is the UWL permeability of the drug. Since the different membrane types were placed in the same side-by-side diffusion cell, the *P*_UWL_ and the thickness of the UWL (*h*_UWL_) are the same for all types of membrane when using the donor and acceptor media, and the same stirring speed.

The unbound fraction (*f*_u_) can be calculated as the equilibrium solubility of the plain buffer (*S*_buffer_) divided by the solubility measured in the presence of HP-β-CD (S_CD_) (Equation (4)).



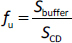

(4)


In this case,



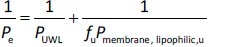

(5)




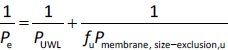

(6)


where *P*_membrane, lipophilic, u_ and *P*_membrane, size-exclusion, u_ are the permeability of unbound drug molecules for PAMPA and size-exclusion membrane, respectively.

*P*_membrane, lipophilic, u_ at each pH can be calculated as Equation (7):



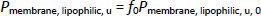

(7)


Membrane permeability in the presence of HP-β-CD (*P*_membrane, lipophilic_) can be calculated considering the free fraction of the drug (Equation (8)):



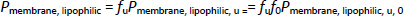

(8)


where *f*_0_ is the unionized fraction of a drug at a certain pH (see Table 1), and *P*_membrane, lipophilic, u_ is the permeability of unbound and unionized drug molecules.

*P*_UWL_ at each pH and stirring speed can be calculated as Equation (9) in the case of pure buffer and Equation (10) in the presence of HP-β-CD:



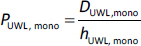

(9)




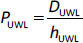

(10)


where *D*_UWL_ is the apparent diffusion coefficient of the drug through the unstirred water layer in the presence of HP-β-CD and *h*_UWL_ is the apparent unstirred water layer thickness in the presence of HP-β-CD.

The diffusion coefficient of the monomer (*D*_UWL, mono_) and the drug-HP-β-CD complex (*D*_UWL, complex_) was calculated at 37 °C using Equation (11) [9]:



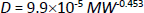

(11)


where *MW* is the molecular weight of the monomer or the drug-HP-β-CD complex.

The apparent diffusion coefficient of the drug through the unstirred water layer in the presence of HP-β-CD can be calculated from the diffusion coefficient of the monomer and the complex following Equation (12):



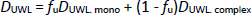

(12)


### Phase solubility study of CAR-HP-β-CD complex

For the investigation of the complexation, an excess amount of crystalline CAR (5 mg Form I or Form II) was added to 10 mL of pH 10 Prisma buffer containing 0, 2.5, 5, 10, 15, 20 mg mL^-1^ HP-β-CD, respectively. The resulting solutions were stirred at 250 rpm for 6 h and then sedimented for 18 h as described in the equilibrium solubility measurement protocol [47,48]. The samples were continuously thermostated at 37 °C. After sedimentation, the concentration of CAR was measured using a UV probe connected to Rainbow μDISS Profiler (Pion Inc, Billerica, MA, USA) at 292 to 300 nm with the second derivative method. In all cases, the excess solids from the solutions were sampled and subjected to differential scanning calorimetry (DSC) with Mettler ToledoDSC 3+ (STARe Evaluation Software [49], 25 to 150 °C, 10 °C min^-1^) and Raman spectroscopy measurements with PerkinElmer System 2000 NIR FT-Raman (Spectrum v5 0.1 software [50], Raman laser power: 500 mW, accumulations: 64, resolution: 4 cm^-1^, Raman shift: 3500 to 100 cm^-1^).

### Membrane transport of HP-β-CD

With μFLUX apparatus (Pion Inc., Billerica MA, USA), 20 mL of Prisma buffer containing 15 mg mL^-1^ HP-β-CD and 50 μg mL^-1^ CAR was placed on the donor side, and 20 mL of pure Prisma buffer was placed on the acceptor side. The stirring speed was kept at 250 rpm, and the CAR concentration of the two solutions was monitored at 37 °C using UV probes connected to Rainbow μDISS Profiler (Pion Inc, Billerica, MA, USA) at 292 to 300 nm with the second derivative method. The transport of HP-β-CD across different membranes (1 kDa and 6 kDa size exclusion membrane and lipophilic membrane) was investigated using the Buvari-Barcza [51] method. For this purpose, CD concentrations were measured in the acceptor solution. A phenolphthalein stock solution of 3.75 mM dissolved in 96 % by volume ethanol was diluted tenfold with distilled water, and a 4 mM Na_2_CO_3_ solution was prepared. In a 250 mL volumetric flask, 20 mL of aqueous phenolphthalein solution was mixed with 25 mL of 4 mM Na_2_CO_3_ solution, and the flask was made up to the mark with distilled water. The pH of the Buvari-Barcza solution was adjusted to 10.5 with Na_2_CO_3_. Every 2 hours for 8 hours after adding 1000 μL of acceptor solution to 10 mL of Buvari-Barcza solution, the absorbance of the solution was measured at 550 nm with Rainbow UV probes. 1000 μL of donor solution was also withdrawn every two hours to ensure that the same amount of fluid was in the μFLUX apparatus on the donor and acceptor sides.

### *In vitro* side-by-side diffusion cell assays

To perform *in vitro* side-by-side diffusion cell assays, the μFLUX (Pion Inc., Billerica MA, USA) apparatus was used. Artificial membranes selected to be a size-exclusion regenerated cellulose membrane (MWCO 6 and 1 kDa, 1.54 cm^2^) and a lipophilic membrane (0.45 μm pore size PVDF sheet 1.54 cm^2^ impregnated with 25 μL *n*-dodecane). Before measurement, the size-exclusion membranes were soaked in distilled water overnight. Measurements were performed using pH 10 Prisma buffer and pH 10 Prisma buffer containing 15 mg mL^-1^ HP-β-CD. The measurements were performed in two arrangements for each membrane type, as shown in Table 2.

**Table 2.** *In vitro* side-by-side diffusion cell setups

Donor side	Acceptor side
pH 10 Prisma buffer	pH 10 Prisma buffer
pH 10 Prisma buffer + HP-β-CD	pH 10 Prisma buffer

18 mL of the appropriate buffer was pipetted to both the donor and acceptor sides. Then, various amounts of 3 mg mL^-1^ methanol stock solution for the drug were pipetted to the donor side (to reach a target concentration between 2.5 and 20 μg mL^-1^ in pH 10 Prisma buffer and 2.5 and 50 μg mL^-1^ in pH 10 Prisma buffer with HP-B-CD). The exact amount of pure methanol was added to the donor and acceptor side and the maximum methanol concentration was kept under 1.57 % to minimize the solvent effect on the solubility and permeability of the API. Both cells were stirred at 37 °C using a magnetic stirrer at 250 rpm for 1 minute, and then the stirring speed was set to either 250 rpm or 0 rpm until the end of the measurements. Drug concentrations at the donor and acceptor sides were monitored in real-time using UV probes with Rainbow apparatus (Pion Inc., Billerica MA, USA). A second derivative method was used between 292 and 300 nm to evaluate the spectra, and the *P*_app_ was calculated [9] according to Equation (1).

## Results and discussion

### Determination of the limiting step of membrane transport for lipophilic and size-exclusion membranes

**Figure 2.**
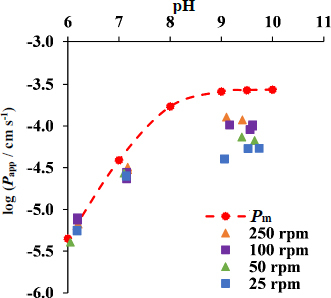
Apparent permeability-pH profile of CAR measured using lipophilic membrane

In case of the lipid membrane, there is a fundamental difference in diffusion in lipophilic and aqueous environment; therefore, the determination of the limiting step of the transport is critical in case of the lipophilic membrane. Therefore, the permeability-pH profile was measured, and the stirring speed dependence was also tested for the lipophilic membrane. Figure 2 shows the pH dependence of the apparent permeability of CAR measured using the *n*-dodecane membrane at the stirring rates tested. At pH 5, no drug permeation through the membrane was observed at any of the stirring rates after 24 hours. In the pH range below the *pK*_a_ of the active substance (p*K*_a_ = 7.78 [36,37]), the slope of the curves is around +1. The permeability of CAR is very low at pH 5 and 6, as CAR is overwhelmingly ionized in solution. At this range, the apparent permeability curves closely approximate the membrane permeability curve (log *P*_m_). As the pH is increased, the molecule with a weak basic character shows increasing permeability, with the curves flattening at pH ranges higher than the p*K*_a_. The apparent permeability curves are shifted from the membrane permeability curve and are already skewed before the p*K*_a_ value is reached. Figure 2 shows that the transport is membrane-limited at pH 6 and 7 (pHs lower than p*K*_a_). At higher pH values (pH 9.5 and 10), where the drug is mostly present in neutral form, the transport becomes UWL-limited.

The results of the stirring rate study also support that conclusion, since at pH 6 and 7, there was no significant effect found on the apparent permeability, while at higher pHs, the apparent permeability, therefore, the UWL was significantly reduced by increasing the stirring speed.

In the case of the lipophilic membrane, the flux and the apparent permeability results were obtained from the initial linear phase of the acceptor curves in each case. The Gutknecht method [42-45] was used to determine the permeability of CAR at all stirring rates. The average of the membrane permeability of the neutral unbound drug (*P*_membrane, lipophilic, u, 0_) was found to be 2.89 10^-4^±0.523010^-4^ cm s^-1^ (log *P*_membrane, lipophilic, u, 0_ = = -3.54 ± 0.08). The *P*_UWL_, *P*_m_, *D*_UWL_ and *h*_UWL_ values were calculated using Equations (2) to (12) and are presented in Table S1 (Supplementary material).

**Figure 3.**
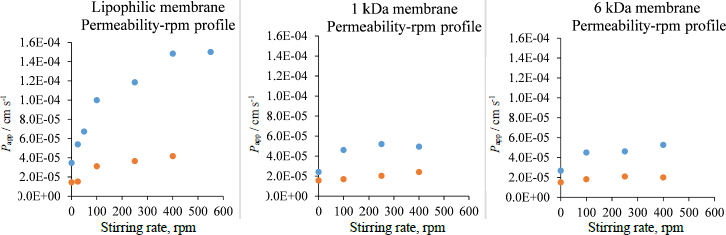
Apparent permeability-rpm profiles for lipophilic, 1 and 6 kDa size-exclusion membranes at 37 °C, pure pH 10 Prisma buffer with blue dots and pH 10 Prisma buffer containing 15 mg mL^-1^ HP-β-CD on the donor side with orange dots

In contrast, in the case of a size-exclusion membrane, both the UWL and the membrane permeation mean aqueous diffusion. However, these two aqueous environments may differ significantly due to the drug's molecular hydration and potential interaction with the filter material. There are practical limitations in determining the limiting step of transport for size-exclusion membranes: since these membranes cannot differentiate between ionized and neutral molecules, due to the absence of lipophilic domain, the permeability shows no pH dependence (*P*_app_ = 5.9 ± 0.8 cm s^-1^ at pH 6 and *P*_app_ = 5.2 ± 0.7 cm s^-1^ at pH 10 in case of 1 kDa MWCO membrane). Therefore, standard methods, such as permeability-pH profiling, are not suitable for calculating the size of the UWL or determining the limiting step of transport. Only stirring speed dependence can be tested (Figure 3), which proves the existence of the UWL, but according to Sugano [10,52], the insensitiveness of apparent permeability to the stirring speed increment does not prove that the transport is membrane-limited.

While the direct approach was not found to measure the *P*_UWL_ for the size-exclusion membranes, using the same apparatus, it can be assumed that the UWL permeabilities and the thickness of the UWL are the same for lipophilic and size-exclusion membranes when using the same donor, acceptor media and stirring speed. In a non-stirred environment, where the UWL is the thickest, and *P*_UWL_ is very close to *P*_app_, (see Table S2), in the case of all 3 membranes in the plain buffer, similar *P*_app_ values could be measured. These results support the assumption that the *P*_UWL_ is the same for different membranes at the given experimental conditions.

Considering the apparent permeability-pH profile (Figure 2) and the *P*_UWL_ and *P*_m_ values in Tables S1 and S2, pH 10 media was chosen for the *in vitro* side-by-side diffusion cell assays to create UWL-limited transport conditions when a lipophilic membrane is used in the absence of HP-β-CD.

### Phase solubility study of CAR - HP-β-CD complex in pH 10 Prisma buffer

**Figure 4.**
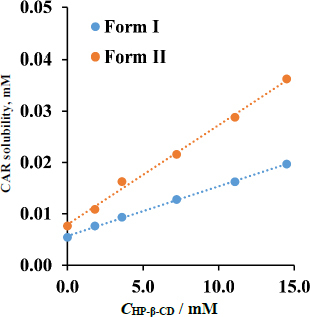
Phase-solubility diagram of CAR-HP-β-CD complex, pH 10 Prisma buffer, 37 °C

Figure 4 shows the phase solubility diagram of CAR's two polymorphic modifications (Form I and Form II). A linear correlation (Form I: *R*^2^=0.9987; Form II: *R*^2^=0.9952) between the active substance's equilibrium solubility and the solution's HP-β-CD content was found for both Form I and Form II modifications. This indicates that the phase solubility is linear at the range of HP-β-CD concentrations studied, suggesting that the drug forms a 1:1 stoichiometric complex with HP-β-CD [53-55].

Based on the thermograms obtained from the differential scanning calorimetry (DSC) measurements of the solid excess (see Supporting Information, Figure S1), we can conclude that the melting point of the samples taken after the equilibrium solubility measurement is the same as that of the corresponding reference materials. There is no difference between the Raman spectra (see supporting information Figure S2) of the sample and the reference materials. Thus, it can be concluded that both polymorphic modifications (Form I and Form II) of carvedilol were sufficiently stable under the measurement conditions and no solvent-mediated polymorphic transformation or amorphisation occurred.

Assuming a 1:1 complexation, the equilibrium constant of the complex (*K*_1:1_) and the complexation efficiency (CE) were determined based on the solubility of the pure active compound (*S*_0_) and the slope of the lines (Table 3).

**Table 3.** Complex stability constant (*K*_1:1_) and complexation efficiency (CE) of CAR- HP-β-CD complex in pH 10 Prisma buffer, 37 °C

CAR polymorph	*S*_0_ / μM	Slope	*K*_1:1_ / M^-1^	CE
Form I	5.412	9.645910^-4^	178.4	0.9654010^-3^
Form II	7.627	1.934110^-3^	254.1	1.938110^-3^

The effect of the solid phase is also responsible for the difference in the efficiency of the complexation of Form I and Form II modifications. In the equilibrium solubility measurements, it was found that the thermodynamically stable Form I has a lower solubility than Form II, and accordingly, the complexation efficiency is lower in the presence of the more stable form.

From the results obtained in the phase-solubility measurement (Figure 3 and Table 34), it can be concluded that HP-β-CD forms a rather stable 1:1 stoichiometric complex with the model API; therefore, HP-β-CD was chosen as a model solubilizing additive with known hydrodynamic size [56] (1-2 nm) for the *in vitro* side-by-side diffusion cell assays, including size-exclusion membranes.

**Table 4.** Thermodynamic solubility of CAR polymorphs in pH 10 Prisma buffer with and without the addition of 15 mg mL^-1^ HP-β-CD, 37 °C

	Form I		Form I + HP-β-CD		Form II		Form II + HP-β-CD	
Equilibrium thermodynamic solubility μg mL^-1^	2.15	±0.23	6.69	±0.54	3.37	±0.21	10.64	±0.87

Since the phase-solubility diagram showed a linear relationship between the HP-β-CD concentration and CAR solubility, the concentration of the solubilizing additive was chosen based on a known HP-β-CD containing marketed solution [57] and the recommendation to take the solution with a glass of water. The thermodynamic solubility results with the chosen 15 mg mL^-1^ HP-β-CD concentration are presented in Table 4.

From the results obtained in the thermodynamic solubility measurement, the solubility of both polymorphs is about three times higher in the presence of 15 mg mL^-1^ HP-β-CD (Table 4).

### Membrane transport of HP-&-CD

**Figure 5.**
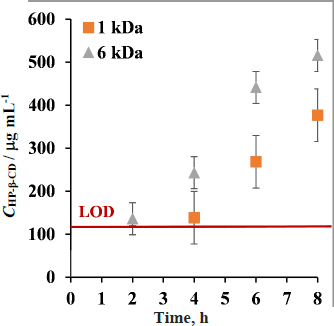
Membrane transport of HP-β-CD at 250 rpm and 37 °C with μFlux apparatus applied lipophilic membrane (was not detectable at concentrations above the LOD), 1 kDa size-exclusion membrane with orange dots, 6 kDa size-exclusion membrane with gray dots, (1.54 cm^2^ membrane surface), using Buvari-Barcza [51] method

To investigate how stable the lipophilic membrane is in the presence of 15 mg mL^-1^ HP-β-CD in the donor compartment and whether HP-β-CD can permeate the size-exclusion membrane, *in vitro* side-by-side diffusion cell assays were carried out with the solubilizing agent and HP-β-CD concentration was detected on the acceptor side of the different membranes. HP-β-CD was detected under the LOD in the acceptor compartment in the case of the lipophilic membrane, so it can be concluded that CD does not cause significant structural damage in the lipophilic layer for 8 hours (Figure 5). The CD is detected increasingly on the acceptor side, reaching 516 μg mL^-1^ concentration in 8 hours in the case of a 6 kDa size-exclusion membrane (meaning 1.075 μg cm^-2^ min^-1^ flux value) and 377 μg mL^-1^ concentration in the case of a 1 kDa size-exclusion membrane (meaning 0.785 μg cm^-2^ min^-1^ flux value), showing that HP-β-CD was able to diffuse across both size-exclusion membranes.

**Figure 6.**
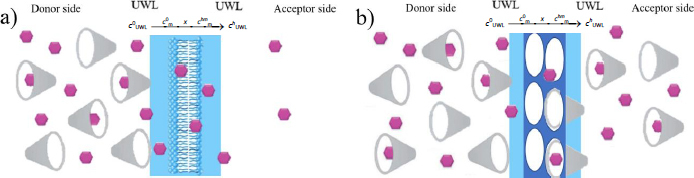
Schematic drawing of membrane transport in case of a) lipophilic membrane b) size-exclusion membrane (API is represented with pink hexagons, while the cyclodextrin is with grey cones). *c*^0^_m_ and *c^h^*^m^_m_ are the concentrations of the uncharged form of the solute within the membrane at the two water–membrane boundaries, *x* is the width of the membrane

Based on the results, the two different scenarios were depicted in Figure 6. In the case of the lipophilic membrane, only the drug is able to permeate the membrane; the solubilizing agent and the drug-cyclodextrin complex are not. In the case of the size-exclusion membranes (MWCO= 1 kDa and 6 kDa), both the drug and the HP-β-CD and their complex are able to go through.

### *In vitro* side-by-side diffusion cell assays

During the *in vitro* side-by-side dissolution cell assays, the aim was to create UWL-limited transport for the model API in the absence of HP-β-CD. Therefore, pH 10 media was used in both compartments, and the effect of the solubilizing agent on flux was studied with 250 rpm stirring and without stirring. Three different membrane types (PVDF filter impregnated with *n*-dodecane) and two size-exclusion membranes (MWCO = 1 and 6 kDa) were used in the study. The lipophilic membrane was a model for the scenario where the drug is able to permeate the membrane, but the solubilizing agent and the drug-cyclodextrin complex are not. While in the case of the size-exclusion membrane, both the drug and the HP-β-CD and their complex are able to go through (see the section on membrane transport of HP-β-CD).

**Figure 7.**
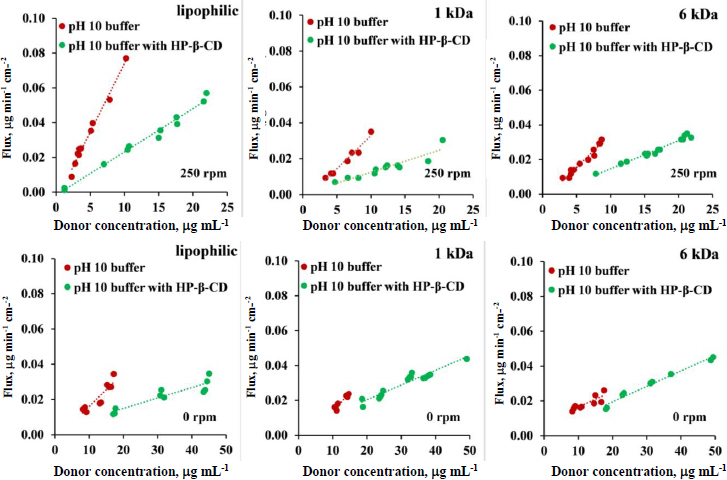
Flux *vs.* donor concentration curves in pH 10 buffer with 250 rpm stirring rate and with 0 rpm stirring rate, in case of lipophilic membrane, 6 kDa and 1 kDa size-exclusion membranes. No HP-β-CD present on either side of the membrane with red dots, addition of HP-β-CD to donor side with green dots

During the side-by-side diffusion cell assays, in most cases, supersaturated solutions were created in the donor compartment both with and without CD present (The donor compartment is considered supersaturated if the concentration exceeds the thermodynamic solubility: 2.15 μg mL^-1^ for the plain buffer and 6.69 μg mL^-1^ for the HP-β-CD containing buffer). Precipitation was monitored in real-time (see Supporting Information, Figure S3). During flux calculation, a time interval was chosen where the donor side was stable, and no precipitation was observed. The flux values obtained from *in vitro* side-by-side diffusion cell assays were plotted as a function of CAR concentration in the donor compartment (Figure 7). The diagrams clearly show that the highest fluxes in absolute value can be achieved by using a lipophilic membrane, 250 rpm stirring, and no solubilizing agent. Changing the membrane to a size-exclusion membrane or leaving the set-up without stirring causes the flux values to decrease significantly.

The effect of solubilizing agent was evaluated by comparing the line of the plain buffer (red) and the buffer with HP-β-CD (green) with a homogeneity of slopes test in all 6 cases depicted in Figure 7. The result of the statistical analysis (Table 5) shows that in the case of 250 rpm stirring for all 3 membranes, the solubilizing additive causes the slope of the red and green lines to differ significantly. The slope is directly proportional to the permeability, meaning that in the case of the stirred configuration, the addition of HP-β-CD causes a significant decrease in the permeability values. On the other hand, in the case of the non-stirred set-up for the size-exclusion membranes, the addition of CD only causes a smaller effect in the slopes, therefore, in the *P*_app_ values.

**Table 5.** Homogeneity of slopes test results for flux-donor concentration curves in pH 10 buffer with 250 rpm stirring rate and with 0 rpm stirring rate, in case of lipophilic membrane, 6 kDa and 1 kDa size-exclusion membranes.

	*p*-value for 250 rpm	p-value for 0 rpm
lipophilic	0.000000	0.000481
1 kDa	0.000033	0.012039
6 kDa	0.000000	0.962032

**Figure 8.**
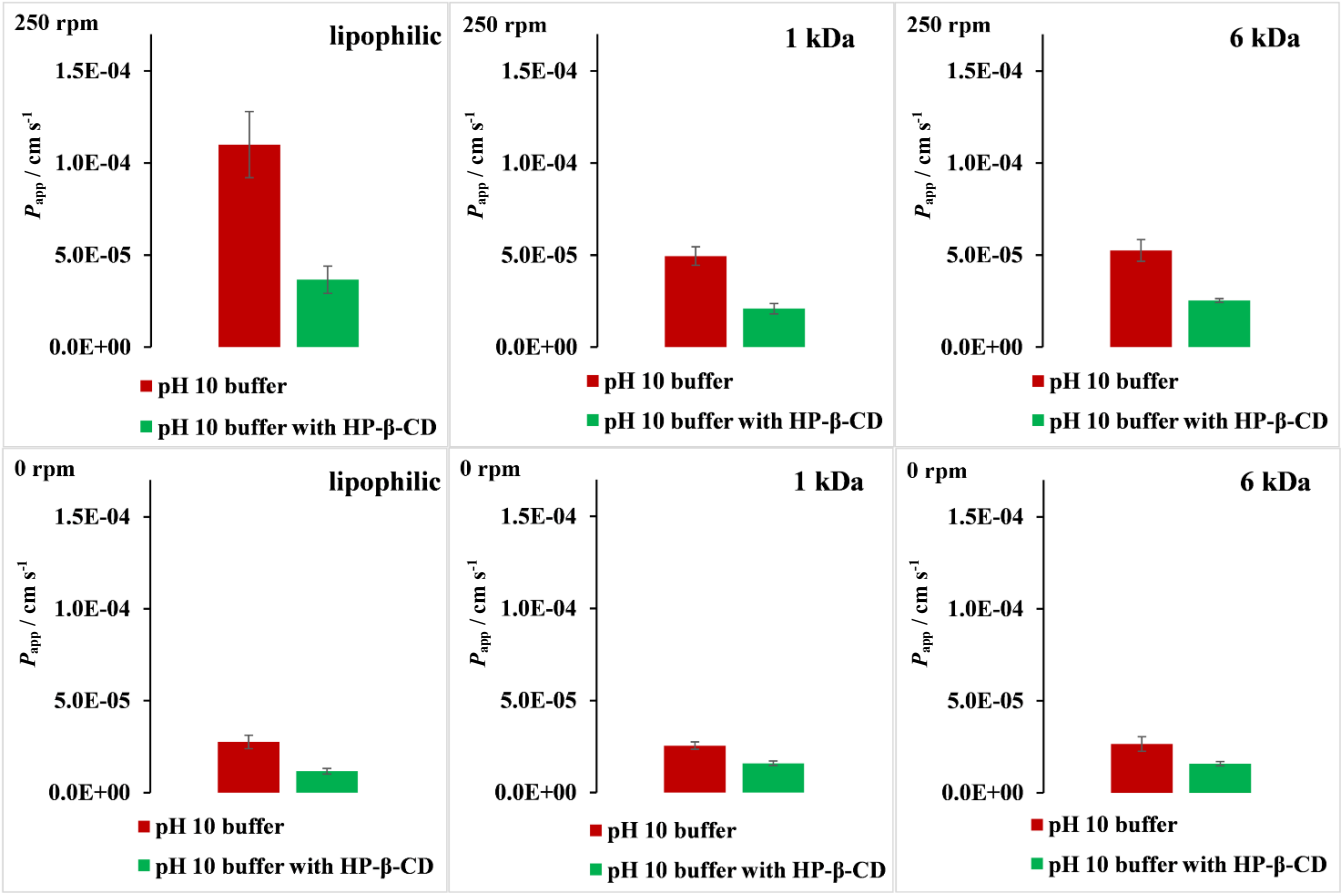
Apparent permeability values in pH 10 buffer with 250 rpm stirring rate and with 0 rpm stirring rate, in case of lipophilic membrane, 6 kDa and 1 kDa size-exclusion membranes. No HP-β-CD present on either side of the membrane, with red, addition of HP-β-CD to the donor side with green

To evaluate the effect of all factors (membrane type, stirring speed, and the presence of solubilizing additive), first, the permeability values were calculated from flux and donor concentration data (see Equation (1)). Apparent permeability (*P*_app_), data are presented in Figure 8. Statistical analysis (3way ANOVA factorial design) showed that all three factors have a significant effect on the apparent permeability. In the case of the different membrane types, the lipophilic membrane showed significantly different *P*_app_ values (*p* < 0.00001), whereas no significant difference could be observed between the two size-exclusion membranes with different MWCOs. The addition of HP-β-CD significantly decreased the *P*_app_ value (*p*<0.00001), demonstrating the solubility-permeability interplay [26,27,58,59]. By introducing sufficient stirring (250 rpm) the *P*_app_ values significantly increased in all cases (*p* <0.00001), which results agree with literature data.

Moreover, the between effects were also evaluated as part of the statistical analysis. All three between-effects were found to significantly influence the *P*_app_ value (see Table S2). Interestingly, the between-effect diagrams showed that in the presence of HP-β-CD in the donor compartment, the introduction of stirring causes a significantly smaller increase in the *P*_app_ value than in the case of the plain buffer. These diagrams also showed that in the presence of CD, the differences between the lipophilic and size-exclusion membranes got smaller in terms of *P*_app_ value. In a non-stirred environment, where the UWL is the thickest, these differences completely disappeared, and in the case of all 3 membranes, similar *P*_app_ values could be measured.

Statistical analysis (3way ANOVA factorial design) was carried out for the *P*_m_ and *P*_UWL_ values calculated from P_app_ (Table S2). The results of the analysis showed that both main factors have a significant effect on the UWL permeability (*P*_uwl_): stirring speed and the presence of HP-β-CD. The membrane type was found not significant due to the assumption that *P*_UWL_, and *h*_UWL_ is the same for different type of membranes using the same buffer composition and stirring speed.

In the case of *P*_m_ data analysis, the results showed that the effect of membrane type and CD was found to be significant. The stirring speed was found to be non-significant, which is consistent with the calculation of *P*_m_. *P*_m_ was found significantly higher in the case of lipophilic membranes than in the case of the size-exclusion membranes, which is reasonable considering that the lipophilic drug diffuses faster in a lipophilic environment. There was no significant difference between the *P*_m_ values measured for the different MWCO membranes. Placing HP-β-CD in the donor compartment caused the *P*_m_ value to decrease in all membrane types.

In conclusion, using a non-stirred environment caused the absolute *P*_app_ values to be similar in the case of all three membranes. However, since non-stirring significantly reduces the effect of solubilizing agents for all three membranes, stirring is essential to observe excipient effects reliably. Therefore, stirring remains crucial for assessing the impact of formulation additives on apparent permeability and for the comparison of different drug formulations containing the same API.

### Mathematical description of membrane transport in UWL limited case

**Figure 9.**
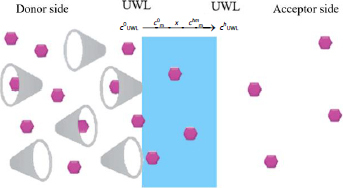
Simplified transport model for UWL limited cases

In this section of the study, the aim was to mathematically describe the transport through the UWL-membrane-UWL system in a general manner, without specifying the type of membrane between the two UWL layers or whether the transport-limiting step is access through the UWL. Therefore, we assumed that the transport through the membrane can be neglected, and only a double-size UWL serves as a barrier for the API to diffuse from the donor to the acceptor compartment (Figure 9). Later in the article, the applicability of such a simplified model will be discussed.

### Transport of the pure API through the UWL

The transport of the pure API through the UWL is most commonly described by Fick’s first law of diffusion (Equation (13)):



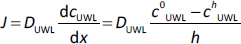

(13)


where *J* is the flux, *D*_UWL_ is the diffusion coefficient through the UWL, *c*^0^_UWL_ and *c^h^*_UWL_ are the concentration of non-ionized molecules on both sides of the UWL and *h* is the thickness of the water layer.

Considering the partitioning between the UWL and the bulk of the donor solution and acceptor solution, Equations (14) and (15):



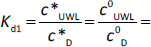

(14)




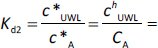

(15)


where *K*_d1_ and *K*_d2_ are partition coefficients for steady state, *c*_D_ and *c*_A_ are concentrations on the donor and the acceptor side, * is the property at saturation. Based on Equations (14) and (15) *c*^0^_UWL_ and *c^h^*_UWL_ can be expressed as:



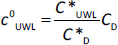

(16)




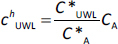

(17)


If Equations (16) and (17) are substituted into Equation (13), then Equation (18) is obtained:



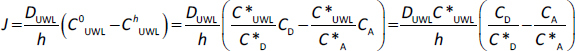

(18)


*D*_UWL_, *h*, C*_D_ and C*_A_ are constants specific to the API.

### Transport of an API in the presence of solubilizing additive

The starting equation is Equation (18).

*D*_UWL_ can be expressed as shown in Equation (12) [60,61]:



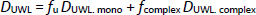

(12)


where *f*_u_ and *f*_complex_ (*f*_u_ + *f*_complex_ = 1) are the fractions of the unbound monomer and complexed API, respectively. The *f*_u_ can be estimated as:



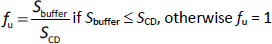

(4)


where *S*_CD_ is the solubility in CD-containing media and *S*_buffer_ is the solubility in the corresponding buffer without CD.

Based on Equation (18), Equations (12) and (4), flux can be expressed as shown in Equation (19):



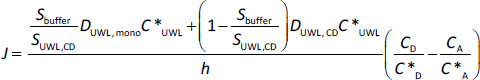

(19)


Considering that C*_UWL_= *S*_UWL, CD_ flux can be expressed as shown in Equation (20):



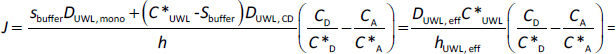

(20)


Where *D*_UWL, eff_ is the effective diffusion coefficient through the UWL and *h*_UWL, eff_ is the effective thickness of the UWL in the presence of HP-β-CD. From Equation (20) it can be concluded that the driving force of membrane transport in case of UWL limited transport condition is the difference between the supersaturation ratio (SSR, defined as the ratio of the drug concentration present in solution to its thermodynamic solubility measured in exactly the same media) in the donor and acceptor compartment. This difference is directly proportional to the flux, and the coefficient of proportionality (*B*_UWL_) is (*D*_UWL, eff_ C*_UWL_) / *h*_UWL, eff_.

### Mathematical description of membrane transport in membrane membrane-limited case

A concentration-based mathematical description was previously published for membrane membrane-limited case in the case of lipophilic and also size-exclusion membranes [22,62].

The main assumption of this model is that the solubilizing agent cannot permeate the membrane; only the free drug is able to pass through, Equation (21).



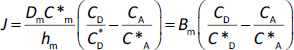

(21)


where *B*_m_ is the coefficient of proportionality, *D*_m_ is the diffusion coefficient through the membrane, *C*_m_ is the concentration of the drug in the membrane and * is the property at saturation, *h*_m_ is the membrane thickness.

### General mathematical description of membrane transport

Based on the membrane and UWL limited cases, a general model can be described, where both the UWL and membrane permeation are considered as consecutive processes. The main assumption of this model is that the complexing agent and the drug-CD complex cannot permeate the membrane. Similarly to permeability values (Equation (2)), a reciprocal relation can be made between the effective B factor and the B factor for the UWL and the membrane (Equation (22)).



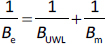

(22)


Therefore, flux can be expressed as Equation (23):



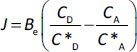

(23)


where *B*_e_ is the effective coefficient of proportionality.

### The application of the general mathematical model to experimental flux data for lipophilic membranes and size-exclusion membranes

Based on the results of Table S2, the limiting step of the transport for the lipophilic membrane can change from UWL to the membrane when HP-β-CD is present. This observation is in agreement with the previously published observation from Dahan and Miller [46]. Therefore, the general model (Equation (23)) was applied to the flux dataset of lipophilic membranes. The primary assumption of this model is that the complexing agent and the drug-CD complex cannot permeate the membrane, which has been proven to be the case for a lipophilic membrane (see Figure 5). Figure 9 shows the flux plotted as a function of the difference in SSR. The slopes of the lines equal the coefficient of proportionality. Homogeneity of slopes tests with the sample size 8-12 were carried out to see if the line of the plain buffer (red) and the HP-β-CD containing buffer (green) differ (Table 6). According to the statistical analysis, the null hypothesis of parallel lines cannot be rejected in the case of lipophilic membranes for both 0 rpm and 250 rpm.

**Table 6.** Homogeneity of slopes test results for flux-SSR curves in pH 10 buffer with 250 rpm stirring rate and with 0 rpm stirring rate, in case of lipophilic membrane, 6 and 1 kDa size-exclusion membranes

	*p*-value for 250 rpm	*p*-value for 0 rpm
Lipophilic	0.149329	0.839690
1 kDa	0.169113	0.205616
6 kDa	0.000028	0.000000

For a size-exclusion membrane with 1 kDa MWCO, the general model was also applied (Figure 9); however, the assumption that the complexing agent is not crossing the membrane is not true, since a small amount of HP-β-CD was observed at the acceptor side (Figure 5). According to the results of the homogeneity of slopes test for 1 kDa MWCO membrane (Table 6), the null hypothesis of parallel lines cannot be rejected in the case of 0 rpm and 250 rpm.

In case of size-exclusion membrane with 6 kDa MWCO significant difference can be seen in the slopes (see *p*-values in Table 6 and also additional statistical information on homogeneity of slopes test in supplement Tables S3 to S14) therefore the null hypothesis is rejected at both 0 rpm and 250 rpm, showing that the amount of CD crossing the 6 kDa size-exclusion membrane (Figure 5) truly makes a significant deviance from the mathematical model that only considers the drug crossing through the membrane.

When comparing the applicability of the model for the 3 membranes, a tendency can be observed in the flux-SSR diagrams. The results of the lipophilic membrane show almost perfect parallel lines, while in the case of the size-exclusion membranes, a slightly higher slope is produced in the case of 1 kDa MWCO membrane, and an even higher slope was seen in the case of the 6 kDa MWCO membrane in the presence of the HP-β-CD. In the case of the 6 kDa membranes, the transport of CD through the membrane is faster (see Figure 5) than in the case of the 1 kDa membrane; therefore, the deviation from the parallel line is also bigger. This observation implies that the deviation from the parallel lines is due to the failure of the main assumption of the model, namely that solubilizing agent and drug-CD complexes do not go through the membrane. Therefore, the increase in flux at a certain SSR value due to the addition of HP-β-CD in the donor compartment can be attributed to the flux generated from the drug-CD complex in the case of size-exclusion membranes.

In conclusion, experimental data supports that in case of the lipophilic and 1 kDa size-exclusion membrane the true driving force of membrane transport is the difference is SSR (Equation (23)), when the membrane dividing the two UWL layers only lets the drug permeate or the permeation of the solubilizing additive is quite slow, so it does not affect the transport of the API substantially. Only in the case of the 6 kDa membrane can a significant deviation be observed from the general model that only considers the drug crossing through the membrane due to the amount of CD crossing the 6 kDa size-exclusion membrane.

**Figure 10.**
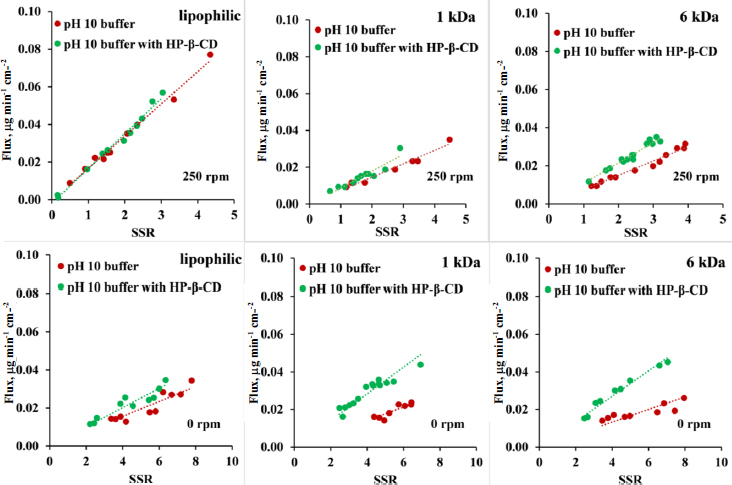
Flux *vs.* SSR curves in pH 10 buffer with 250 rpm stirring rate and with 0 rpm stirring rate, in case of lipophilic membrane, 6 and 1 kDa size-exclusion membranes. No HP-β-CD present on either side of the membrane with red dots, addition of HP-β-CD to donor side with green dots.

## Conclusions

The comparative study of lipophilic and size-exclusion membranes in *in vitro* side-by-side diffusion cell assays in the presence of cyclodextrins presents a comprehensive evaluation of the mechanisms underlying drug transport. The research focused on carvedilol as the model drug and hydroxypropyl-beta-cyclodextrin as the model solubilizing agent, aiming to understand the differences in permeability and solubility when using these two types of membranes.

The solubility measurements indicated that HP-β-CD effectively increased the solubility of CAR for both polymorph forms, suggesting its role in enhancing drug solubility. The phase solubility diagram further confirmed that CAR forms a 1:1 stoichiometric complex with HP-β-CD. The transport of HP-β-CD through the lipophilic membrane was negligible. In contrast, it was substantial through the size-exclusion membrane, highlighting a key difference between the two types of membrane. *In vitro* side-by-side diffusion cell assays provided valuable insight into how HP-β-CD influences drug transport across these membranes. The presence of HP-β-CD on the donor side generally reduced permeability due to increased drug solubility and decreased free drug concentration; with this, the Solubility-Permeability Interplay was well displayed in the case of both membrane types. In conclusion, using a non-stirred environment caused the absolute *P_app_* values to be similar in the case of all three membranes. However, since non-stirring significantly reduces the effect of solubilizing agents for all three membranes, stirring is essential to observe excipient effects reliably. Therefore, stirring remains crucial for assessing the impact of formulation additives on permeability and for the comparison of different drug formulations containing the same API.

The supersaturation ratio, defined as the ratio of the drug concentration present in solution to its thermodynamic solubility measured in exactly the same media. was found to be the driving force of membrane transport due to a concentration-based mathematical description. This mathematical description was also supported with experimental data for lipophilic and 1kDa MWCO size-exclusion membranes with the transport of carvedilol and HP-β-CD, when the permeation of the solubilizing additive is quite slow, so it does not affect the transport of the API substantially.

Overall, the similar driving force across lipophilic membranes and via 1 kDa MWCO size-exclusion membranes provides a robust platform for assessing drug permeability. This can streamline the drug development process by reducing the reliance on more complex and challenging cell-based models, offering a more practical approach to pharmaceutical research and development. Future research could refine these findings by exploring a wider range of drugs and solubilizing agents, ultimately enhancing the predictive power and applicability of size-exclusion membranes in pharmaceutical sciences.

## Supplementary material

Additional data are available at https://pub.iapchem.org/ojs/index.php/admet/article/view/2753, or from the corresponding author on request.


