# Supplementary material for: Comparison of lipophilic and size-exclusion membranes: the effect of stirring and cyclodextrin in the donor compartment
Source: ADMET DMPK. 2025 Jul 5;13(4):2753. doi: 10.5599/admet.2753 (PMC12335304; doi:10.5599/admet.2753)
Supplement: Supplementary file 1 [file ADMET-13-2753-S1.pdf]

Open Access : ISSN : 1848-7718

http://www.pub.iapchem.org/ojs/index.php/admet/index

Supplementary material to

Comparison of lipophilic and size-exclusion membranes: the effect of stirring and cyclodextrin in the donor compartment

Petra Tőzsér[1], Szabina Kádár[1], Edina Szabó[1], Máté Dobó[2], Gergő Tóth[2], György T. Balogh[2,3,4], Péter Sóti[5], Bálint Sinkó[6], and Enikő Borbás[1]

[1]*Department of Organic Chemistry and Technology, Faculty of Chemical Technology and Biotechnology, Budapest University of Technology and Economics, 3 Műegyetem Quay, H-1111, Budapest, Hungary*
[2]*Department of Pharmaceutical Chemistry, Faculty of Pharmaceutical Sciences, Semmelweis University, 9 Hőgyes Endre Street., H-1092, Budapest, Hungary*
[3]*Center for Pharmacology and Drug Research & Development, Semmelweis University, 26 Üllői Street., H-1085, Budapest, Hungary*
[4]*Department of Chemical and Environmental Process Engineering, Faculty of Chemical Technology and Biotechnology, Budapest University of Technology and Economics, 3 Műegyetem Quay., H-1111, Budapest, Hungary*
[5]*Lavet Pharmaceutical Ltd., 6 Batthyány Street., H-2143, Kistarcsa, Hungary*
[6]*Pion Inc., Billerica, 10 Cook Street, Massachusetts 01821, USA*

ADMET & DMPK **13(4)** (2025) 2753; https://doi.org/10.5599/admet.2753

Differential scanning calorimetry curves and Raman spectras in case of CAR Form I and Form II are presented on Figures S1 and S2, respectively.

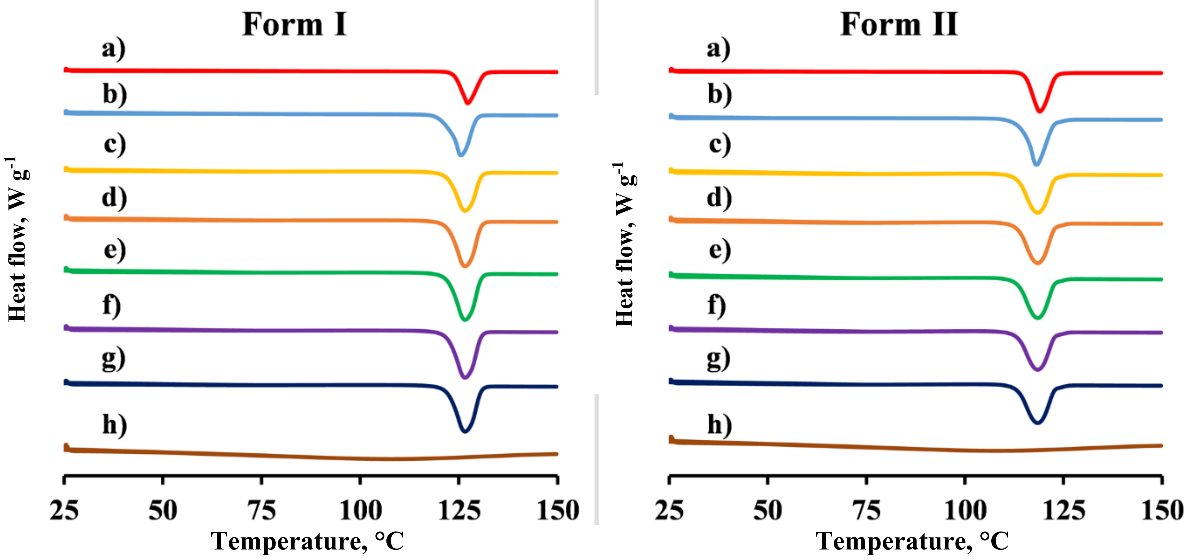

Figure S1. Differential scanning calorimetry curves in case of CAR Form I and Form II. a) Reference polimorpf b) polimorph in pH 10 Prisma buffer c) polimorph in pH 10 Prisma buffer containing 2.5 mg mL^{-1}HP-β-CD d) polimorph in pH 10 Prisma buffer containing 5 mg mL^{-1}HP-β-CD e) polimorph in pH 10 Prisma buffer containing 10 mg mL^{-1}HP-β-CD f) polimorph in pH 10 Prisma buffer containing 15 mg mL^{-1} HP-β-CD g) polimorph in pH 10 Prisma buffer containing 20 mg mL^{-1} HP-β-CD h) HP-β-CD reference

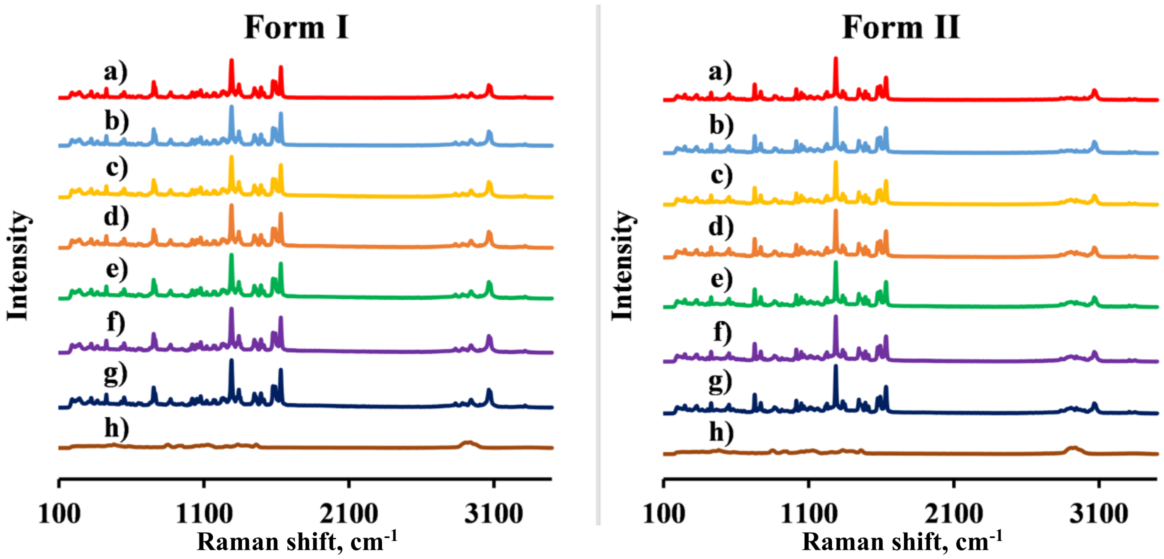

Figure S2. Raman spectras in case of CAR Form I and Form II. a) Reference polimorpf b) polimorph in pH 10 Prisma buffer c) polimorph in pH 10 Prisma buffer containing 2.5 mg mL^{-1} HP-β-CD d) polimorph in pH 10 Prisma buffer containing 5 mg mL^{-1} HP-β-CD e) polimorph in pH 10 Prisma buffer containing 10 mg mL^{-1} HP-β-CD f) polimorph in pH 10 Prisma buffer containing 15 mg mL^{-1} HP-β-CD g) polimorph in pH 10 Prisma buffer containing 20 mg mL^{-1} HP-β-CD h) HP-β-CD reference.

The *in vitro* side-by-side diffusion cell assays were carried out at 5 different pH buffers and 4 stirring speeds and the change of concentrations on both the donor and acceptor sides was monitored in real-time (Figure S3).

In the case of a), the neutral form of the active substance is present in the solution at pH 10. The uncharged molecules pass relatively easily through the lipophilic membrane. For the 250 and 100 rpm stirring, CAR was detected on the acceptor side 20-25 minutes after the measurement was started, while for 25 rpm it was detected after only 40 minutes. Furthermore, the precipitation of the active substance from the super-saturated solution, a common phenomenon with poor water solubility of active substances, can be observed in this figure. On the donor side, precipitation is indicated by a sharp change in the concentration curves, while on the acceptor side, a break is observed in the section corresponding to the time of precipitation.

In case b) pH 9.5, the neutral form is also present in the solution in predominant amounts, at this pH, the time for the drug to pass through at each stirring rate was almost identical to that described in case a).

In the experiment at pH 7, the neutral and ionized forms of the active substance are present in nearly equal amounts. In this case, it was observed that transport across the membrane is slower. Even at the 250 rpm stirring rate, the drug was detected in the acceptor cell after more than 2 hours, and at lower stirring rates, the drug was transferred across the membrane after 3 hours.

At the pH conditions of case d) shown in Figure S3, a significant fraction of the total amount of active substance in the solution is already in ionized form. Under these conditions, the measurement time was significantly increased, with the first detection of CAR on the acceptor side after more than 13 hours.

Further increasing the proportion of the ionized form present in the solution, a side-by-side diffusion cell assay was also performed at pH 5. However, no permeation of the drug through the membrane was observed at any of the stirring rates after 24 hours.

CC BY

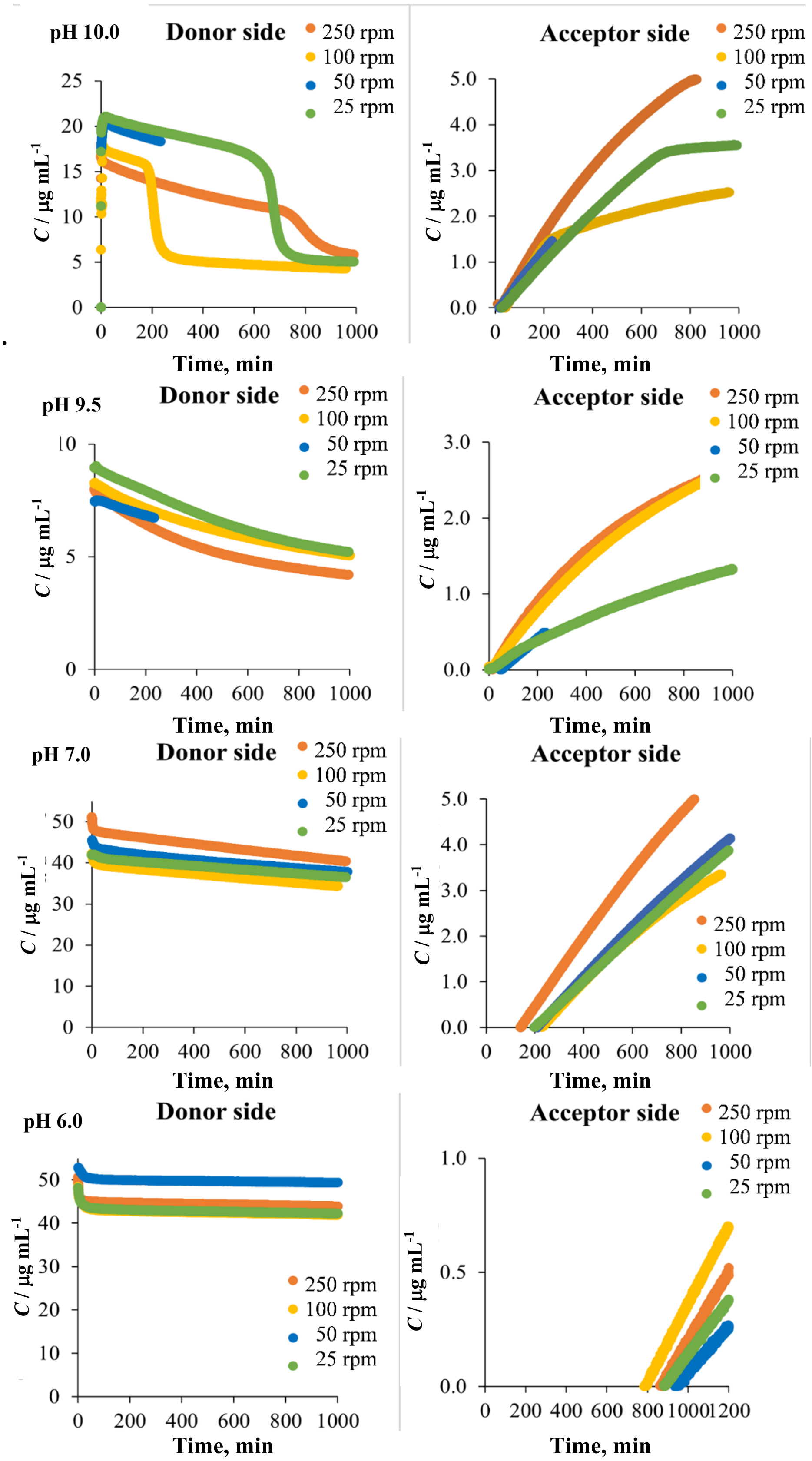

Figure S3. *In vitro* UWL study concentrations of CAR at the donor and acceptor side at different pH conditions and stirring speeds (250 rpm with orange, 100 rpm with yellow, 50 rpm with blue, 25 rpm with green)

The P_{UWL}, P_m, D_{UWL} and h_{UWL} values calculated using Equations (2) to (12) are presented in Table S1, and P_{app}, P_{UWL} and P_m values from the *in vitro* side-by-side permeation assay are presented in Table S2.

Table S1. Results of the UWL thickness measurement in case of lipophilic membrane with different pH values and stirring rates

Stirring rate, rpm	Theoretical pH	Measured pH	Flux, µg cm^{-2} min^{-1}	Time interval, min	P_{app} / 10^{-5} cm s^{-1}	P_m / 10^{-5} cm s^{-1}	P_{UWL} / 10^{-5} cm s^{-1}	D_{UWL} / 10^{-5} cm^2 s^{-1}	h_{UWL} / µm
250	10	9.41	0.103	100-200	11.90	28.73	20.32	0.65	320
	9.5	9.10	0.052	100-200	12.80	28.32	23.36	0.65	280
	7	7.17	0.085	400-500	3.21	3.96	16.96	0.65	380
	6	6.20	0.018	900-1000	0.67	0.46	-	0.65	-
100	10	9.61	0.097	100-200	10.00	28.73	15.34	0.65	420
	9.5	9.16	0.046	100-200	10.30	28.32	16.19	0.65	400
	7	7.15	0.062	400-500	2.79	3.96	9.45	0.65	690
	6	6.19	0.020	900-1000	0.80	0.46	-	0.65	-
50	10	9.65	0.077	100-200	6.73	28.73	8.79	0.65	740
	9.5	9.40	0.031	100-200	7.41	28.32	10.04	0.65	650
	7	7.10	0.068	200-300	2.73	3.96	8.79	0.65	740
	6	6.05	0.012	1000-1100	0.41	0.46	-	0.65	-
25	10	9.74	0.070	50-100	5.40	28.73	6.65	0.65	980
	9.5	9.06	0.020	100-200	4.03	28.32	4.70	0.65	1390
	7	7.14	0.060	400-500	2.54	3.96	7.09	0.65	920
	6	6.18	0.014	1100-1200	0.56	0.46	-	0.65	-

-at pH 6 there was no significant difference in the P_m and P_e results, therefore P_{UWL} values were not calculated
--if $P_{UWL} < P_m$ then the system tends towards UWL limitation, these cases have green background
---if $P_{UWL} > P_m$ then the system tends towards membrane limitation, these cases have blue background

Table S2. Summary table of P_{app}, P_{UWL} and P_m values from the *in vitro* side-by-side permeation assay

		Lipophilic membrane		1 kDa size-exclusion membrane		6 kDa size-exclusion membrane	
		pH 10 buffer	pH 10 buffer with HP-β-CD	pH 10 buffer	pH 10 buffer with HP-β-CD	pH 10 buffer	pH 10 buffer with HP-β-CD
0 rpm	P_{app} / 10^{-5} cm s^{-1}	2.76	1.17	2.55	1.59	2.66	1.58
	P_e / 10^{-5} cm s^{-1}	n.a.	3.76	n.a.	n.a.	n.a.	n.a.
	P_m / 10^{-5} cm s^{-1}	28.73	9.23	15.30	2.12	20.35	2.10
	P_{UWL} / 10^{-5} cm s^{-1}	3.05	6.35	3.06	6.35	3.06	6.35
	D_{UWL} / 10^{-5} cm^2 s^{-1}	0.65	0.44	0.65	0.44	0.65	0.44
	h_{UWL} / µm	2130	690	2130	690	2130	690
100 rpm	P_{app} / 10^{-5} cm s^{-1}	9.99	3.12	4.61	1.70	4.51	1.83
	P_e / 10^{-5} cm s^{-1}	n.a.	7.16	n.a.	n.a.	n.a.	n.a.
	P_m / 10^{-5} cm s^{-1}	28.73	9.23	6.60	1.80	6.39	1.94
	P_{UWL} / 10^{-5} cm s^{-1}	15.320	3.184	1.532	3.184	1.532	3.184
	D_{UWL} / 10^{-5} cm^2 s^{-1}	0.65	0.44	0.65	0.44	0.65	0.44
	h_{UWL} / µm	430	140	430	140	430	140
250 rpm	P_{app} / 10^{-5} cm s^{-1}	10.99	3.66	4.94	2.09	5.26	2.54
	P_e / 10^{-5} cm s^{-1}	n.a.	7.41	n.a.	n.a.	n.a.	n.a.
	P_m / 10^{-5} cm s^{-1}	28.73	9.23	6.82	3.17	7.44	4.27
	P_{UWL} / 10^{-5} cm s^{-1}	17.80	37.60	17.80	37.60	17.80	37.60
	D_{UWL} / 10^{-5} cm^2 s^{-1}	0.65	0.44	0.65	0.44	0.65	0.44
	h_{UWL} / µm	360	120	360	120	360	120
400 rpm	P_{app} / 10^{-5} cm s^{-1}	14.84	4.17	4.96	2.42	5.28	2.01
	P_e / 10^{-5} cm s^{-1}	n.a.	8.07	n.a.	n.a.	n.a.	n.a.
	P_m / 10^{-5} cm s^{-1}	28.73	9.23	5.92	2.51	6.38	2.07
	P_{UWL} / 10^{-5} cm s^{-1}	30.69	63.92	30.69	64.91	30.69	64.91
	D_{UWL} / 10^{-5} cm^2 s^{-1}	0.65	0.44	0.65	0.44	0.65	0.44
	h_{UWL} / µm	210	70	210	70	210	70

if $P_{UWL} < P_m$ then the system tends towards UWL limitation, these cases have green background; if $P_{UWL} > P_m$ then the system tends towards membrane limitation, these cases have blue background; if there was no significant difference found between the P_m and P_{UWL} values then beige background was used.

CC BY

A summary of the slope homogeneity test for flux and SSR diagrams is shown in Figures S3 to S14 (*df* - degree of freedom. SE - standard error).

Table S3. Univariate tests of significance for flux (250 rpm lipophilic), overparameterized model, Type III decomposition

Effect	SS	*df*	MS	*F*	*p*	Partial ε^2	Non-centrality	Observed power (α=0.05)
Intercept	0.000008	1	0.000008	1.589	0.224545	0.085464	1.589	0.221463
Additive	0.000003	1	0.000003	0.589	0.453272	0.033496	0.589	0.112047
SSR	0.006833	1	0.006833	1427.477	0.000000	0.988231	1427.477	1.000000
Additive-SSR	0.000011	1	0.000011	2.281	0.149329	0.118304	2.281	0.296866
Error	0.000081	17	0.000005					

Table S4. Parameter estimates (250 rpm lipophilic), overparameterized model

Effect	Level of effect	Column	Comment (B/Z/P)	Flux param.	Flux SE	Flux *t*	Flux *p*	-95.00 % Conf. lmt	+95.00 % Conf. lmt	Flux β	Flux SE β
Intercept		1		-0.000483	0.001387	-0.34803	0.732088				
Additive	Donor	2	Biased	-0.001503	0.001959	-0.76757	0.453272	-0.154223	0.071942	-0.041141	0.053598
Additive	Pure	3	Zeroed	0							
SSR		4		0.017129	0.000626	27.37012	0	0.886652	1.034775	0.960719	0.035101
Additive-SSR	1	5	Biased	0.001426	0.000944	1.51031	0.149329	-0.033876	0.20456	0.085342	0.056506
Additive-SSR	2	6	Zeroed	0							

Table S5. Univariate tests of significance for flux (250 rpm 1kDa size-exclusion), overparameterized model, Type III decomposition

Effect	SS	*df*	MS	*F*	*p*	Partial ε^2	Non-centrality	Observed power (α=0.05)
Intercept	0	1	0	0.0038	0.951851	0.00027	0.0038	0.050377
Additive	0	1	0	0.0117	0.915376	0.000835	0.0117	0.051169
SSR	0.000773	1	0.000773	176.2197	0.00000	0.926401	176.2197	1.000000
Additive-SSR	0.000009	1	0.000009	2.1023	0.169113	0.130557	2.1023	0.271885
Error	0.000061	14	0.000004					

Table S6. Parameter estimates (250 rpm 1 kDa size-exclusion), overparameterized model

Effect	Level of effect	Column	Commen t (B/Z/P)	Flux param.	Flux SE	Flux *t*	Flux *p*	-95.00 % Conf. lmt	+95.00 % Conf. lmt	Flux β	Flux SE β
Intercept		1		0.000063	0.001979	0.03164	0.975208				
Additive	Donor	2	Biased	-0.000290	0.002679	-0.1082	0.915376	-0.402068	0.363451	-0.019309	0.17846
Additive	Pure	3	Zeroed	0.000000							
SSR		4		0.0037276	0.000696	10.46005	0.00000	0.770244	1.167588	0.959916	0.09263
Additive-SSR	1	5	Biased	0.001784	0.001231	1.44992	0.169113	-0.110639	0.572358	0.230859	0.159222
Additive-SSR	2	6	Zeroed	0.000000							

Table S7. Univariate tests of significance for flux (250 rpm 6kDa size-exclusion), overparameterized model, Type III decomposition

Effect	SS	*df*	MS	*F*	*p*	Partial ε^2	Non-centrality	Observed power (α=0.05)
Intercept	0.000002	1	0.000002	1.2289	0.279602	0.052902	1.2289	0.185493
Additive	0	1	0.000000	0.0891	0.768079	0.004035	0.0891	0.059406
SSR	0.001196	1	0.001196	614.7212	0.000000	0.965448	614.7212	1.000000
Additive-SSR	0.000041	1	0.000041	20.9342	0.000148	0.487588	20.9342	0.991978
Error	0.000043	22	0.000002					

Table S8. Parameter estimates (250 rpm 1 kDa size-exclusion), overparameterized model

Effect	Level of effect	Column	Comment (B/Z/P)	Flux param.	Flux SE	Flux *t*	Flux *p*	-95.00 % Conf. lmt	+95.00 % Conf. lmt	Flux β	Flux SE β
Intercept		1		-0.000793	0.001147	-0.69151	0.496479				
Additive	Donor	2	Biased	-0.000585	0.001959	-0.29856	0.768079	-0.299578	0.224177	-0.0377	0.126275
Additive	Pure	3	Zeroed	0.00000							
SSR		4		0.007785	0.000413	18.87091	0.000000	0.711034	0.886613	0.798824	0.042331
Additive-SSR	1	5	Biased	0.003524	0.00077	4.57539	0.000148	0.31281	0.831477	0.572143	0.125046
Additive-SSR	2	6	Zeroed	0.00000							

Table S9. Univariate tests of significance for flux (0 rpm lipophilic), overparameterized model, Type III decomposition

Effect	SS	*df*	MS	*F*	*p*	Partial ε^2	Non-centrality	Observed power (α=0.05)
Intercept	0	1	0	0.0321	0.860095	0.002001	0.0321	0.053264
Additive	0.000011	1	0.000011	1.3498	0.262353	0.077799	1.3498	0.194105
SSR	0.000895	1	0.000895	106.0251	0.000000	0.868879	106.0251	1.000000
Additive-SSR	0	1	0	0.0423	0.83969	0.002635	0.0423	0.054303
Error	0.000135	16	0.000008					

Table S10. Parameter estimates (0 rpm lipophilic), overparameterized model

Effect	Level of effect	Column	Comment (B/Z/P)	Flux param.	Flux SE	Flux *t*	Flux *p*	-95.00 % Conf. lmt	+95.00 % Conf. lmt	Flux β	Flux SE β
Intercept		1		-0.003008	0.003454	-0.87107	0.396597				
Additive	Donor	2	Biased	0.005213	0.004487	1.161806	0.262353	-0.266856	1.022858	0.362046	0.311623
Additive	Pure	3	Zeroed	0.00000							
SSR		4		0.004425	0.000614	7.205766	0.000002	0.684667	1.255437	0.970052	0.134622
Additive-SSR	1	5	Biased	0.00018	0.000877	0.205509	0.83969	-0.560195	0.680533	0.060169	0.292638
Additive-SSR	2	6	Zeroed	0.00000							

Table S11. Univariate tests of significance for flux (0 rpm 1 kDa size-exclusion membrane), overparameterized model, Type III decomposition

Effect	SS	*df*	MS	*F*	*p*	Partial ε^2	Non-centrality	Observed power (α=0.05)
Intercept	0	1	0	0.01798	0.894736	0.000946	0.01798	0.051861
Additive	0.000012	1	0.000012	2.53103	0.128128	0.117553	2.53103	0.326923
SSR	0.00038	1	0.00038	77.5831	0.000000	0.803278	77.5831	1.000000
Additive-SSR	0.000008	1	0.000008	1.71767	0.205616	0.082908	1.71767	0.23796
Error	0.000093	19	0.000005					

Table S12. Parameter estimates (0 rpm 1 kDa size-exclusion), overparameterized model

Effect	Level of effect	Column	Comment (B/Z/P)	Flux param.	Flux SE	Flux *t*	Flux *p*	-95.00 % Conf. lmt	+95.00 % Conf. lmt	Flux β	Flux SE β
Intercept		1		-0.004514	0.005835	-0.77363	0.448671				
Additive	Donor	2	Biased	0.0039859	0.006197	0.643228	0.528028	-0.315024	0.594291	0.139634	0.216961
Additive	Pure	3	Zeroed	0.000000							
SSR		4		0.004360	0.001055	4.131061	0.000568	0.335689	1.02518	0.680434	0.164712
Additive-SSR	1	5	Biased	0.001524	0.001163	1.310597	0.205616	-0.123019	0.530877	0.203929	0.155891
Additive-SSR	2	6	Zeroed	0.000000							

Table S13. Univariate tests of significance for flux (0 rpm 6 kDa size-exclusion membrane), overparameterized model, Type III decomposition

Effect	SS	*df*	MS	*F*	*p*	Partial ε^2	Non-centrality	Observed power (α=0.05)
Intercept	0.000046	1	0.000046	12.7338	0.002565	0.443164	12.7338	0.917332
Additive	0.000019	1	0.000019	5.2031	0.036583	0.245395	5.2031	0.57265
SSR	0.000835	1	0.000835	229.5317	0.000000	0.934835	229.5317	1.00000
Additive-SSR	0.000241	1	0.000241	66.2241	0.000000	0.80541	66.2241	1.00000
Error	0.000058	16	0.000004					

Table S14. Parameter estimates (0 rpm 6 kDa size-exclusion), overparameterized model

Effect	Level of effect	Column	Comment (B/Z/P)	Flux param.	Flux SE	Flux *t*	Flux *p*	-95.00 % Conf. lmt	+95.00 % Conf. lmt	Flux β	Flux SE β
Intercept		1		0.008029	0.002007	4.00124	0.001029				
Additive	Donor	2	Biased	-0.006262	0.002745	-2.28104	0.036583	-0.65801	-0.024019	-0.340014	0.148061
Additive	Pure	3	Zeroed	0							
SSR		4		0.001927	0.000368	5.24113	0.000061	0.162055	0.52155	0.341802	0.085215
Additive-SSR	1	5	Biased	0.004471	0.000549	8.13782	0	0.861501	1.468456	1.164979	0.143156
Additive-SSR	2	6	Zeroed	0							

CC BY
